# Handgrip Strength Predicts Functional Decline at Discharge in Hospitalized Male Elderly: A Hospital Cohort Study

**DOI:** 10.1371/journal.pone.0069849

**Published:** 2013-07-25

**Authors:** Carmen García-Peña, Luis C. García-Fabela, Luis M. Gutiérrez-Robledo, Jose J. García-González, Victoria E. Arango-Lopera, Mario U. Pérez-Zepeda

**Affiliations:** 1 Unidad de Investigación Epidemiológica y en Servicios de Salud, Area de Envejecimiento. Centro Médico Nacional Siglo XXI, Instituto Mexicano del Seguro Social, México, Distrito Federal, México; 2 Departamento de Geriatría, Instituto de Seguridad Social del Estado de México y Municipios, Estado de México, México; 3 Instituto Nacional de Geriatría, México, Distrito Federal, México; 4 Hospital Regional 1, Instituto Mexicano del Seguro Social, Querétaro, México; Cardiff University, United Kingdom

## Abstract

Functional decline after hospitalization is a common adverse outcome in elderly. An easy to use, reproducible and accurate tool to identify those at risk would aid focusing interventions in those at higher risk. Handgrip strength has been shown to predict adverse outcomes in other settings. The aim of this study was to determine if handgrip strength measured upon admission to an acute care facility would predict functional decline (either incident or worsening of preexisting) at discharge among older Mexican, stratified by gender. In addition, cutoff points as a function of specificity would be determined. A cohort study was conducted in two hospitals in Mexico City. The primary endpoint was functional decline on discharge, defined as a 30-point reduction in the Barthel Index score from that of the baseline score. Handgrip strength along with other variables was measured at initial assessment, including: instrumental activities of daily living, cognition, depressive symptoms, delirium, hospitalization length and quality of life. All analyses were stratified by gender. Logistic regression to test independent association between handgrip strength and functional decline was performed, along with estimation of handgrip strength test values (specificity, sensitivity, area under the curve, etc.). A total of 223 patients admitted to an acute care facility between 2007 and 2009 were recruited. A total of 55 patients (24.7%) had functional decline, 23.46% in male and 25.6% in women. Multivariate analysis showed that only males with low handgrip strength had an increased risk of functional decline at discharge (OR 0.88, 95% CI 0.79–0.98, p = 0.01), with a specificity of 91.3% and a cutoff point of 20.65 kg for handgrip strength. Females had not a significant association between handgrip strength and functional decline. Measurement of handgrip strength on admission to acute care facilities may identify male elderly patients at risk of having functional decline, and intervene consequently.

## Introduction

A number of adverse outcomes have been identified in the elderly following hospitalization; however, functional decline – one of these adverse outcomes – is a hallmark of the development of dependency in this age group [1]. In particular, functional decline after hospitalization is a common adverse outcome in older adults, primarily due to immobilization, polypharmacy, isolation, delirium and pressure sores [2]. Moreover, in this context, functional decline, which is defined as appearance or worsening of limitations in performingactivities of daily living, also reflects the negative interaction between elderly health status (acute illness) and an adverse environment (hospitalization) with the onset of limitations in daily activities (new onset functional decline) or the worsening of preexisting ones [3]. Therefore, an accurate tool for predicting functional decline (either new onset or worsening of preexisting functional decline) would be useful for identifying subjects requiring a more thorough geriatric assessment and intervention [4]–[6].

There are a number of tools that aim to predict functional decline at discharge in hospitalized elderly. Nevertheless, most of these tools are burdensome to apply and subjective, with a lack of comparability and standardization [7]. In contrast, handgrip strength (HS) is a physical performance test that requires little training and only requires a few minutes, with results comparable between populations (mainly kilograms or Newtons). Rather than be a specific test (e.g. hand force) it reflects global health of the elder individual, becoming similar to a “vital sign” (8). In addition, HS is widely used in the elderly for different purposes and has been shown to be predictive of adverse outcomes in other settings [8]–[11]. A recent systematic review [12] analyzed 45 studies of HS as a predictor of adverse outcomes (mortality, functional decline, institutionalization); some of the articles included younger individuals or discussed patients with specific health problems, such as arthritis, pneumonia, or hip fracture. Low HS was a consistent predictor of death (most frequent adverse outcome tested) among all these diverse populations. Eight of the studies also reported a positive correlation between HS and future functional decline. However, the definitions of functional decline and the durations of follow-up varied between the studies. Only two studies [13], [14] evaluated functional decline in an acute care setting at patient discharge; however, they excluded participants with fewer than 6 days of hospitalization or cognitive impairment, and one of these reports was from a specialized care setting (rehabilitation unit). On the other hand, some issues regarding the prognostic value of HS in diverse populations have risen, such as differences between genders. Hicks et al. reported recently that in the InCHIANTI study, HS was not predictive mobility decline in women [15].

A comprehensive geriatric assessment is the gold standard of geriatric care; it has been demonstrated to provide benefits in a number of health problems in elderly. Nevertheless, assessing every elderly person admitted to an acute care unit is difficult [5], [16], particularly in a general hospital setting with a shortage of geriatricians and an increasing rate of hospitalization. The utilization of HS assessments might be supported due to the simplicity and capability for accurate measurements of this test. Moreover, HS could help to identify patients predicted to need a complete evaluation at an early stage of hospitalization.

The aims of this study were to test whether the basal HS of elderly patients admitted to an acute care unit was independently associated with functional decline at discharge - either incident or worsening of preexisting functional decline - and to determine the HS cutoff point as a function of specificity, with an emphasis in gender differences.

## Methods

### Ethics Statement

The study was reviewed and accepted by the "Comisión Nacional de Investigación Científica de la Coordinación de Investigación en Salud, del Instituto Mexicano del Seguro Social” (National Commission of Scientific Research of the Health Research Commission of the Mexican Social Security Institute), which includes the approval of the Ethics and Methodological sub commissions with the registry number: 2005-785-170. All procedures in this research complied with the Helsinki Declaration; and all subjects signed informed consent. The informed consent procedure was performed by the interviewers, and included a thorough explanation of the study, in the presence of the study subject, and two independent witnesses; emphasizing the absolute freedom to make the decision to enter or not, and ensuring that this decision would not affect any of the attention given to the subject. Due to the setting (hospital), at least two visits were done in order to make sure that the subjects completely understood the information given. Once this was performed, and if the subject accepted, a copy of a written explanation was handed to the study subject, the interviewer and the witnesses; after which everyone signed an original and a copy (including the interviewer). The study subject kept the original document and hardcopies were archived. Additionally, if the subject during the interview or in the rest of the process of the study felt that he or she did not want to continue, its participation was stopped, reassuring that all the care received will be exactly the same.

### Setting and Subjects

An acute care cohort study was performed in two hospitals of the main health system in Mexico (Instituto Mexicano del Seguro Social). The study was originally planned to determine the effectiveness of a geriatric unit compared to the usual care provided in an internal medicine ward; the study methods are described elsewhere [16]. Briefly, all patients at least 60 years of age who were admitted during a two-year period (2007–2009) to one of three acute care units (2 internal medicine wards and 1 geriatric unit) that fulfilled selection criteria were screened for the fulfillment of the inclusion criteria. The inclusion criteria were the presence of at least one geriatric problem (falls, slow walking speed, fatigue, sorrow, depression, memory deficit or difficulty with instrumental activities or bathing), as assessed at the first visit after admission using a simple dichotomous question (e.g., “Have you had any falls in the last six months?”, answer = yes or no). Patients who were unable to communicate, referred from the intensive care unit, under mechanical ventilation, receiving parenteral nutrition or exhibiting altered consciousness were excluded. A sample was drawn from this cohort, and the size was calculated using a formula for cohort studies [17]; with reported functional trajectories during hospitalization as a reference for the estimation, as reported by Sleiman et al., which considers the relative risk of developing functional decline at discharge in hospitalized elderly. This risk was 4.1 (2.9–5.6) with a power of 80% and an alpha error of 0.05 [18]. The sample size calculation indicated that 197 subjects were needed, to which 20% was added to compensate for subject losses, for a total of 236 subjects required for the sample.

### Procedures

Trained nurses conducted face-to-face interviews and collected clinical data from the subjects’ medical records. Reliability analyses (dependent and target independent variable) indicated a Cronbach’s alpha of 0.871 for the Barthel Index (BI) score and an intraclass correlation coefficient (interrater, two-way mixed effects) of 0.861 for HS (p<0.001 for both tests).

The main outcome is referred to as functional decline and was understood in two fashions: subjects without any difficulty in activities of daily living or an addition of more difficulties to those present at hospitalization. Operationalization of functional decline was done with the BI score; a score 30 points lower at discharge than that registered in the baseline assessment. Also special cases such as a subject with a basal BI score of 25 or lower was considered disabled if the final BI score was zero (in order to avoid floor effects) [16], [18]. A validated Spanish version of the BI was used [19]. From now on we would refer to incident and worsening of functional decline, only as functional decline.

HS was measured during the basal assessment, which was performed within 48 h after admission to the acute care unit. A standardized technique (in a seated position and with the elbow flexed at 90°) and the Exacta™ digital dynamometer (North Coast Medical, Inc., Gilroy, CA, USA) were used [8]; the higher score of three tests performed with the dominant hand was used for the analysis. No *a prior* cutoff points were used; the measurement was assessed as a continuous variable, with kilograms as the unit of measure.

The other variables measured at baseline were considered as confounding factors, including socio demographic characteristics (age, relationship status, number of persons living in the household and number of years of education), health self-perception, instrumental activities of daily living, delirium, quality of life, cognitive function, depressive symptoms, pressure sores and clinical data extracted from medical records. Health self-perception was evaluated as excellent, very good, good, bad or very bad using a Likert scale question. The validated Spanish version of Lawton and Brody’s Instrumental (LBI) Activities of Daily Living scale was used; this scale is composed of eight items, with a lowest possible score of 0 and a highest possible score of 16 [20]. Delirium was assessed with the Spanish version of the Confusion Assessment Method (CAM) [21]. Quality of life was measured with the visual analog scale of the European Quality of Life (VAS EuroQoL), in which subjects rate their quality of life on a 0-to-100-point scale, with the highest score indicating the best possible score [22]. Cognitive function was assessed using the validated Spanish version of the Mini-Mental State Examination (MMSE). This test evaluates memory, orientation in space and time, calculation, language and word recognition; scores range from 0 to 30 points, with lower scores indicating poorer cognitive ability [20]. Depression was assessed using the 30-item Geriatric Depression Scale (GDS 30) [23], [24]. The presence of pressure sores was determined by a thorough visual examination of the skin by the interviewers. The clinical data included the main cause of hospitalization, prescribed medications and comorbidities. In addition, the Acute Physiology and Chronic Health Evaluation II (APACHE II) Score was used, to reliably assess the severity of an acute condition by integrating a series of laboratory values (e.g., sodium levels), vital signs (e.g., heart rate) and clinical items (e.g., neurological status), with a maximum score of 76 points indicating the worst prognosis [21]. The comorbidity burden was measured with the Charlson Index (Ci), with scores ranging from 0 to 37 [22]. In addition, hospitalizations in the geriatric evaluation and management unit (GEMU) or internal medicine ward (IMW) and the length of hospitalization were recorded, as well as in hospital mortality.

### Statistical Analysis

Descriptive statistics of the whole sample with means and standard deviations were performed for the continuous variables, and the absolute and relative frequencies were determined for the nominal variables. Bivariate and multivariate analyses were stratified by gender. Comparisons between the subjects with and without functional decline were performed (within strata) using the t-test for independent samples for continuous variables and a chi-square test for nominal variables. A multiple logistic regression model with functional decline status as a dependent variable was used to test the independent association with HS for each stratum; confounding variables that were significantly different in the bivariate analysis were added to the models. Age, years of education, unit of hospitalization (GEMU vs IMW), length of hospitalization and the LBI, APACHE II and Ci scores were also included in the multiple logistic regression analysis due to their potential interactions with the outcome variable. Those subjects who died during hospitalization were analyzed as with the adverse outcome; nevertheless, they were also analyzed in three other ways (eliminated, as missing data and multiple imputation) in order to see if there were any differences in the association. Regarding the multivariate analyses, the first model was fully adjusted (all the variables), and the second model only included significant variables (stepwise). Finally, if there was a statistically significant independent association between HS and functional decline, receiver operating characteristic (ROC) curves were generated to determine the optimal cutoff point for HS according to specificity. With the identified cutoff points, the sensitivity, specificity, positive predictive value, negative predictive value, positive likelihood ratio, negative likelihood ratio and area under the curve (AUC) were calculated. All the analyses were performed with STATA version 12.0 (StataCorp LP, Texas, USA).

## Results

A total of 223 subjects were followed up during a mean of 10.56 days of hospitalization (see Table 1), having an estimated power for a specificity of 90% of 0.83. Basal information was gathered in the first 6.5 hours on average. Twelve patients died during hospitalization 4 women (3.2%) and 8 men (8.2%). Of the sample, 56.1% (n = 125) were women, and the mean age of all the subjects was 73.31 years (SD 8.27). A total of 53.4% were unmarried, and the mean number of persons living with the elderly was 2.54 (SD 2.18). Regarding the level of education, 12.6% had never attended school, and the mean number of years of education was 5.87 (SD 4.64). From the total sample, 127 subjects were hospitalized in the geriatric unit (57%). The most frequent diagnosis was pneumonia (11.7%, n = 26), followed by coronary heart disease (10.3%, n = 23), chronic obstructive pulmonary disease exacerbation (9.4%, n = 21) and complications of diabetes mellitus (8.1%, n = 18) (Table 1).

**Table 1:** General patient characteristics.

Variable	Total (N = 223)	
**Age in years,** *mean (SD)*	73.31	(8.27)
**Gender,** *n (%)*		
Male	98	(43.94%)
Female	125	(56.06%)
**No partner,** *n (%)*	119	(53.4)
**Number of persons living with the subject,** *mean (SD)*	2.54	(2.18)
**Analphabetism,** *n (%)*	28	(12.6)
**Years of education,** *mean (SD)*	5.87	(4.64)
**GEMU hospitalization,** *n (%)*	127	(57)
**Length of hospitalization in days,** *mean (SD)*	10.56	(15.67)
**Main diagnosis,** *n (%)*		
Pneumonia	26	(11.7)
Ischemic cardiopathy	23	(10.3)
COPD exacerbation	21	(9.4)
Diabetes complications	18	(8.1)
Heart failure	18	(8.1)
Hepatic failure	16	(7.2)
**Number of medications,** *mean (SD)*	3.39	(2.21)
**EuroQoL VAS score,** *mean (SD)*	69.75	(23.48)
**Health self-perception,** *n (%)*		
Excellent	5	(2.3)
Very good	1	(0.5)
Good	53	(24.4)
Bad	117	(53.9)
Very bad	41	(18.9)
**LBI score,** *mean (SD)*	9.93	(5.05)
**Basal BI score,** *mean (SD)*	87.56	(22.17)
**Functional decline,** *n (%)*	55	(24.7)
**GDS 30 score,** *mean (SD)*	9.18	(5.76)
**MMSE score,** *mean (SD)*	21.64	(4.8)
**Pressure sores,** *n (%)*	10	(4.5)
**Delirium,** *n (%)*	13	(5.8)
**APACHE II score,** *mean (SD)*	10.41	(4.17)
**Ci score,** *mean (SD)*	4.93	(2.68)
**HS in kilograms,** *mean (SD)*	15.67	(8.1)
**Mortality,** *n (%)*	12	(5.4)

*Notes:* n = number of subjects, SD = standard deviation, GEMU = Geriatric Evaluation and Management Unit, COPD = chronic obstructive pulmonary disease, EuroQoL VAS = European Quality of Life Visual Analog Scale, LBI = Lawton and Brody Index, BI = Barthel index, GDS 30 = Geriatric Depression Scale of 30 items, MMSE = Mini-Mental Status Examination, APACHE II = Acute Physiology and Chronic Health Evaluation II, Ci = Charlson Index, HS = handgrip strength.

† p<0.05.

The mean BI baseline score was 87.56 (SD 22.17), no subjects had a BI score of zero, and eight had a score of 25 or less (excluding zero). A total of 55 subjects (24.7%) exhibited a reduction in the BI score of at least 30 points at discharge. An overall mean HS of 15.67 kg (SD 8.1) was found; the means were 19.53 kg (SD 8.85) for men and 12.64 kg (SD 5.98) for women (*p*<0.001).

For male subjects, 76.53% did not have functional decline, whereas 23 subjects had functional decline (23.46%). For this group LBI, GDS 30, MMSE, pressure sores and delirium were significantly different between the functional decline groups (more frequent in those with functional decline). In addition, the mean HS was 21.26 kg (SD 8.63) in the group without functional decline and 13.89 kg (SD 7.17) in the functional decline group (*p*<0.001) (see Table 2 for details).

**Table 2:** Bivariate analysis comparing subjects with and without functional decline* stratified by gender.

Variable	Male						Female					
	Without functional decline (n = 75)		With functional decline (n = 23)		Total (n = 98)		Without functional decline (n = 93)		With functional decline (n = 32)		Total (n = 125)	
**Age in years,** *mean (SD)*	71.46	(7.6)	73.65	(9.7)	71.97	(8.14)	74.08	(8.29)	75.12	(8.26)	74.35	(8.26)
**No partner,** *n (%)*	25	(33.33)	7	(30.43)	32	(32.65)	61	(65.59)	26	(81.25)	87	(69.6)
**Number of persons living with the subject,** *mean (SD)*	2.7	(2.48)	3	(1.88)	2.77	(2.3)	2.48	(2.08)	2	(1.88)	2.36	(2.04)
**Analphabetism,** *n (%)*	69	(92)	19	(82.61)	88	(89.8)	78	(83.87)	29	(90.62)	107	(85.6)
**Years of education,** *mean (SD)*	6.76	(4.62)	6.08	(4.01)	6.6	(4.47)	5.12	(4.81)	5.78	(4.4)	5.29	(4.7)
**GEMU hospitalization,** *n (%)*	50	(66.67)	15	(65.22)	65	(66.33)	46	(49.46)	16	(50)	62	(49.6)
**Main diagnosis,** *n (%)*												
Pneumonia	9	(12)	4	(30.77)	13	(13.27)	11	(11.83)	2	(6.25)	13	(10.4)
Ischemic cardiopathy	13	(17.33)	1	(4.35)	1	(14.29)	7	(7.53)	2	(6.25)	9	(7.2)
COPD exacerbation	4	(5.33)	2	(8.7)	6	(6.12)	11	(11.83)	4	(12.5)	15	(12)
Diabetes	8	(10.67)	0	(0)	8	(8.16)	8	(8.6)	2	(6.25)	10	(8)
Heart failure	8	(10.67)	0	(0)	8	(8.16)	7	(7.53)	3	(9.38)	10	(8)
Hepatic failure	4	(5.33)	3	(13.04)	7	(7.14)	8	(8.6)	1	(3.12)	9	(7.2)
**Number of medications,** *mean (SD)*	2.92	(2.27)	3.34	(2.49)	3.02	(2.31)	3.64	(2.06)	3.81	(2.22)	3.68	(2.09)
**EuroQoL VAS score**, *mean (SD)*	69.66	(21.96)	67.61	(25.67)	69.21	(22.7)	71.6	(23.13)	65.8	(26.92)	70.15	(24.15)
**Health self-perception,** *n (%)*												
Excellent	2	(2.74)	1	(4.55)	3	(3.16)	2	(2.17)	0	(0)	2	(1.64)
Very good	0	(0)	0	(0)	0	(0)	1	(1.09)	0	(0)	1	(0.82)
Good	23	(31.51)	4	(18.18)	27	(28.42)	20	(21.74)	6	(20)	26	(21.31)
Bad	37	(50.68)	12	(54.55)	49	(51.58)	53	(57.61)	15	(50)	68	(55.74)
Very bad	11	(15.07)	5	(22.72)	16	(16.84)	16	(17.39)	9	(30)	25	(20.49)
**LBI score**, *mean (SD)*	11.49	(4.7)	8.91	(4.22)	10.88	(4.7)†	9	(5.41)	9.71	(4.59)	9.18	(5.21)
**GDS 30 score,** *mean (SD)*	7.81	(5.21)	10.34	(6.58)	8.4	(5.63)†	9.73	(5.21)	9.96	(5.4)	9.79	(5.82)
**MMSE score**, *mean (SD)*	23.88	(3.97)	19.04	(3.74)	22.74	(4.41)†	21.1	(4.8)	19.81	(5.23)	20.77	(4.41)
**Pressure sores,** *n (%)*	2	(2.67)	4	(17.39)	6	(6.12)†	2	(2.15)	2	(6.25)	4	(3.2)
**Delirium,** *n (%)*	1	(1.33)	4	(17.39)	5	(5.1)†	4	(4.3)	4	(12.5)	8	(6.4)
**APACHE II score**, *mean (SD)*	10.28	(4.24)	10.6	(4.05)	10.35	(4.17)	10.48	(4.18)	10.34	(4.21)	10.44	(4.17)
**Ci score**, *mean (SD)*	4.73	(2.53)	5.6	(3.51)	4.93	(2.8)	4.82	(2.53)	5.21	(2.76)	4.92	(2.59)
**HS in kilograms,** *mean (SD)*	21.26	(8.63)	13.89	(7.17)	19.53	(8.85)†	13.03	(6.06)	11.52	(5.72)	12.64	(5.98)
**Length of hospitalization in days,** *mean (SD)*	7.61	(4.4)	20.95	(6.79)	10.74	(16)†	8.83	(14.08)	14.96	(15.52)	10.4	(14.65)†

*Notes:* n = number, SD = standard deviation, GEMU = Geriatric Evaluation and Management Unit, COPD = chronic obstructive pulmonary disease, EuroQoL VAS = European Quality of Life Visual Analog Scale, LBI = Lawton and Brody Index, GDS 30 = Geriatric Depression Scale of 30 items, MMSE = Mini-Mental Status Examination, APACHE II = Acute Physiology and Chronic Health Evaluation II, Ci = Charlson index, HS = handgrip strength.

* Subjects who died were also considered to have incident functional decline.

† p<0.05.

Female functional decline was present in 25.6% (n = 32) of patients, which was not significantly different compared with the male subjects (*p = *0.714). The mean HS for the group of functional decline was 11.52 kg (SD 12.64) and 13.03 kg (SD 6.06) for those female subjects without functional decline (*p = *0.219). Compared to the male group, HS was significantly different (*p*<0.001). There were no significantly different variables for this group, when comparing those female subjects with and without functional decline (see Table 2 for details).

The fully adjusted multivariate logistic regression model showed that in male subjects, the odds ratio (OR) of HS was 0.87 (95% confidence interval [CI] 0.76–0.96, *p* = 0.01), and the MMSE OR was 0.64 (95% CI 0.49–0.83); number of days of hospitalization had also a significant association, OR 1.25 (95% CI 1.25–1.52), these three variables remained significant in the second model (pseudo-R^2^ = 0.412). For female subjects, only MMSE and basal BI score were significant in the fully adjusted model, with an OR of 0.86 and 1.07, respectively The second model was not performed due to lack of significance in the fully adjusted regression (Table 3).

**Table 3:** Multiple logistic regression models for functional decline stratified by gender.

Gender	Male						Female		
Variable	Model 1 OR (95% CI)		*p*	Model 2 OR (95% CI)		*p*	Model 1 OR (95% CI)		*p*
**HS in kilograms**	0.87	(0.76–0.96)	0.01	0.88	(0.79–0.98)	0.03	0.93	(0.84–1.03)	0.088
**Age in years**	1	(0.9–1.11)	0.977	–		–	1.05	(0.98–1.11)	0.347
**Years of education**	1.03	(0.86–1.24)	0.671	–		–	1.08	(0.96–1.22)	0.177
**GEMU hospitalization**	0.74	(0.14–3.94)	0.734	–		–	1.13	(0.37–3.43)	0.824
**EuroQoL VAS score**	0.99	(0.96–1.03)	0.683	–		–	0.99	(0.97–1.01)	0.46
**Basal Barthel score**	1.07	(0.99–1.17)	0.069	1.05	(0.99–1.11)	0.058	1.07	(1.02–1.12)	0.001
**LBI score**	0.91	(0.72–1.16)	0.471	–		–	1.04	(0.87–1.24)	0.62
**GDS 30 score**	0.95	(0.79–1.13)	0.577	–		–	1.05	(0.96–1.16)	0.248
**MMSE score**	0.644	(0.49–0.83)	0.001	0.65	(0.52–0.81)	<0.001	0.86	(0.75–0.99)	0.041
**Pressure sores**	9.33	(0.01–328.4)	0.679	–		–	12.79	(0.47–347.59)	0.13
**Delirium**	18.65	(0.94–367.99)	0.054	–		–	9.43	(0.53–166.65)	0.125
**APACHE II score**	0.84	(0.7–1.02)	0.094	–		–	1.01	(0.88–1.17)	0.806
**Ci score**	1.15	(0.88–1.51)	0.299	–		–	1.12	(0.9–1.39)	0.309
**Length of hospitalization in days**	1.25	(1.03–1.52)	0.02	1.27	(1.09–1.48)	0.002	1.02	(0.99–1.06)	0.088

*Notes:* OR = odds ratio, HS = handgrip strength, CI = confidence interval, GEMU = Geriatric Evaluation and Management Unit, EuroQoL VAS = European Quality of Life Visual Analog Scale, LBI = Lawton and Brody Index, GDS 30 = Geriatric Depression Scale of 30 items, MMSE = Mini-Mental Status Examination, APACHE II = Acute Physiology and Chronic Health Evaluation II, Ci = Charlson index.

Model 1: fully adjusted model: HS, age, years of education, hospitalization in the GEMU, EuroQoL VAS, LBI score, GDS 30 score, MMSE score, pressure sores, delirium, APACHE II score and Ci score.

Model 2: only significant variables (stepwise).

**Figure 1:**
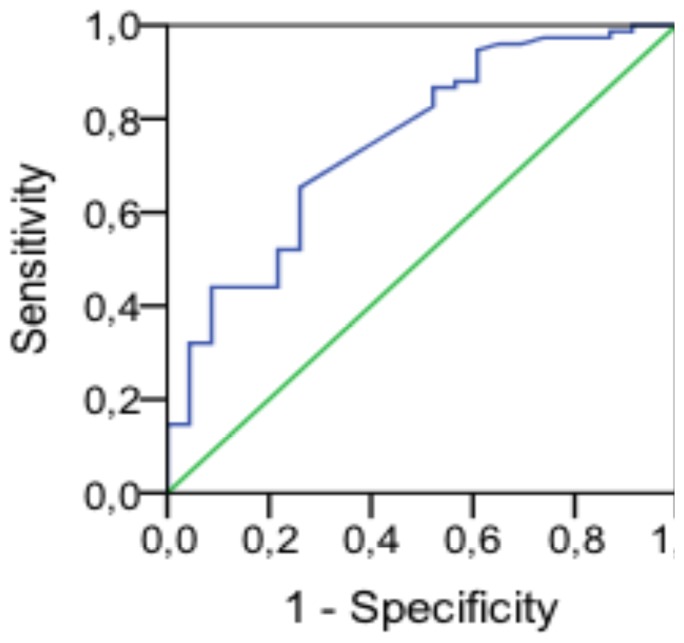
ROC curves for functional decline in function of different cut points of handgrip strength of male subjects.

Finally, the ROC curve identified a cutoff point of 20.65 kg (specificity 91.3%) for male subjects with an area under the curve of 0.712 and (Figure 1,Table 4 and Table 5).

**Table 4:** A 2×2 table with a cut-off point of 20.65 kg for male subjects, showing absolute frequencies; next table shows values derived from this table.

	With functional decline	Without functional decline	Totals
HS <20.65kg	13	6	19
HS >20.65kg	10	69	79
Totals	23	75	98

HS = handgrip strength.

**Table 5:** Sensitivity, specificity, positive predictive value, negative predictive value, positive likelihood ratio, and negative likelihood ratio for handgrip strength, with cut-off points of 20.65 kg for male subjects.

Gender	Male
Sensitivity	56%
Specificity	91.3%
Positive predictive value	68.42%
Negative predictive value	87.34%
Positive likelihood ratio	1.27
Negative likelihood ratio	0.095
AUC	0.751

*Notes:* AUC = area under the curve.

## Discussion

An inverse association was found between HS and functional decline in male subjects; a 1-kg reduction in HS below 20.65 kg in the basal evaluation was associated with a 14.9% increase in the risk of having functional decline at discharge; this was not the case for female subjects, were HS was not significantly different in the bivariate analysis between disabled and non-disabled women. As previously noted, Hicks et al. showed that among community-dwelling elderly, HS is not associated with long-term incident mobility decline [15]; in this study, the prognostic value of HS was lower in women than in men. On the other hand, adjusting for height has shown to diminish this effect and could be a possible explanation for this phenomenon, as showed by Savino et al [25]. Stratifying the analysis for gender could partially adjust for the body size, nevertheless as proposed by Sallinen, further stratification for body mass index and determine cut points accordingly [26]. However other reports have not used an adjustment for body size [8].

Along with the basal MMSE score, HS provides a useful test to predict functional decline among hospitalized elderly male subjects. In accordance with previous studies of hospitalized elderly [12], HS was able to predict functional decline at discharge. In the hospital context, HS has also been shown to accurately predict postsurgical outcomes, survival, institutionalization and long-term (>6 months) incident functional decline [12], [27], [28]. Although comprehensive geriatric assessments have been shown to improve adverse outcomes among hospitalized elderly, some studies have noted that such assessments may not be cost-effective [5], [29]–[31]. The use of HS as a screening tool could allow physicians to focus on patients at higher risk of adverse outcomes; and implement early interventions. This is of particular relevance in settings were specialized health care for the elderly is limited, and could be a part of an incremental geriatric assessment, nevertheless there is need of more research on this topic. In addition, due to the low sensitivity of the test, the need for complete geriatric assessment for misclassified subjects could provide better accuracy with an articulated evaluation of these subjects. Additionally, those misclassified subjects (due to the high rate of false negatives) will benefit from a second assessment, and the intervention could be performed in this one (in contrast to those detected with HS that will receive immediate interventions) [32].

In addition to HS, it appears that both low physical performance and low cognitive performance can predict incident functional decline [7], [33] in a number of different settings. Our study found similar results. However, a low MMSE score could also reflect the presence of incident delirium at admission. As demonstrated by our results, although there was a bivariate association between delirium and functional decline, this association was not confirmed by the multivariate analysis. Although the odds ratios for cognitive impairment in the adjusted multiple regressions were surprisingly high, it is possible that the clinical conditions at admission and/or delays in care could explain these results. Nevertheless, a number of authors have hypothesized that there is an association between frailty, particularly physical frailty, and cognitive impairment [34]. In contrast, other researchers, such as Giampaoli et al., have not found an association between basal cognitive decline and functional decline [10].

Muscle strength could be a global measure of overall health status. Furthermore, a causal relationship between strength and functional decline can be argued, even when the mechanisms involved are unclear [35]. As with vital signs, when certain “functional signs” display abnormal values, clinicians should be encouraged to search for underlying causes in the entire patient rather than focusing on the organ where the functional sign was detected. Along with other signs, HS assessments produce added value in the global evaluation of the elderly. Similar results have been found for other physical performance tests, such as gait speed [36], with respect to their capacity to predict functional decline and other adverse outcomes in diverse settings. However, gait speed is not feasible to assess in hospitalized patients. Instead, grip strength is simple, portable and affordable. Mexico has approximately 370 certified geriatricians to care for more than 10 million people older than 60 [37]. In addition, general hospitals at IMSS face an increasing demand for hospitalization in internal medicine and surgery wards [38]. In settings where there are limited geriatric human resources and elderly care is a challenge, a simple screening test could help clinicians focus on those patients with the worst prognoses.

Along with physical performance tests and the rest of the geriatric assessment, nutritional status evaluation and anthropometry have been shown to be predictor of adverse outcomes, and it would have been useful to adjust HS to these variables [25], [39]. We chose to classify the subjects who died during hospitalization as disabled because death is the worst possible result during hospitalization. In fact, the results did not change substantially when these cases were excluded or included as if they had no functional decline. Although this classification may have caused some bias in the results, it is expected that a large fraction of those who died were disabled [40]. The selection criteria limit the generalization of the results; nevertheless, this fraction of the elderly could benefit from more focused geriatric attention. Moreover, the prognostic value of HS should be tested in a wider population of hospitalized elderly individuals to observe its utility for both “high functioning” and “highly disabled” elderly subjects admitted to acute care. The definition of functional decline could have floor effects in those subjects with the lowest scores; in our sample, only 14 subjects had this condition (6.28%), and all had a BI score of zero in the final assessment. In addition to this well known physical performance test, further research should also include comparisons with newer devices, such as accelerometers, that could provide more information about mobility, as recently demonstrated by Pedersen et al. (particularly in hospitalized elderly), and eventually, these measurements could become the gold standard [41] compared with HS.

Only a few of the studies included in a systematic review by Bohannon reported a follow-up periods that were restricted to the time that the patients were hospitalized. Although a longer follow-up period would have been desirable for our study, limiting the data to the hospitalization period will certainly be useful to hospital clinicians [12].

Finally, we conclude that HS may be useful for detecting male elderly patients older than 60 years who are at risk of functional decline at hospital discharge and could aid in focusing interventions on those with higher risk. Nevertheless, further research aimed to clarify differences between genders in handgrip strength is warranted by our findings.
